# Sol-Gel Derived Tertiary Bioactive Glass–Ceramic Nanorods Prepared via Hydrothermal Process and Their Composites with Poly(Vinylpyrrolidone-Co-Vinylsilane)

**DOI:** 10.3390/jfb11020035

**Published:** 2020-06-01

**Authors:** Dibakar Mondal, Andrei Zaharia, Kibret Mequanint, Amin S. Rizkalla

**Affiliations:** 1 Department of Chemical and Biochemical Engineering, The University of Western Ontario, London, ON N6A 5B9, Canada; dmondal2@uwo.ca (D.M.); kmequani@uwo.ca (K.M.); 2 Bone and Joint Institute, The University of Western Ontario, London, ON N6A 5B9, Canada; 3 Schulich Dentistry, The University of Western Ontario, London, ON N6A 5B9, Canada; azaharia2019@dents.uwo.ca; 4 School of Biomedical Engineering, The University of Western Ontario, London, ON N6A 5B9, Canada

**Keywords:** hydrothermal processing, bioactive glass-ceramics, co-polymer, mechanical properties, composites

## Abstract

Bioactive glass (BG) nanoparticles have wide applications in bone repair due to their bone-bonding and biodegradable nature. In this work, nanometric rod-shaped ternary SiO_2_-CaO-P_2_O_5_ bioactive glass particles were prepared through sol-gel chemistry followed by a base-induced hydrothermal process at 130 °C and 170 °C for various times up to 36 h. This facile, low-temperature and surfactant-free hydrothermal process has shown to be capable of producing uniform nanorods and nanowires. One-dimensional growth of nanorods and the characteristics of siloxane bridging networks were dependent on the hydrothermal temperature and time. Hardened bioactive composites were prepared from BG nanorods and cryo-milled poly(vinylpyrrolidone-co-triethoxyvinylsilane) in the presence of ammonium phosphate as potential bone graft biomaterials. Covalent crosslinking has been observed between the organic and inorganic components within these composites. The ultimate compressive strength and modulus values increased with increasing co-polymer content, reaching 27 MPa and 500 MPa respectively with 30% co-polymer incorporation. The materials degraded in a controlled non-linear manner when incubated in phosphate-buffered saline from 6 h to 14 days. Fibroblast cell attachment and spreading on the composite were not as good as the positive control surfaces and suggested that they may require protein coating in order to promote favorable cell interactions.

## 1. Introduction

Bioactive glasses (BGs) are osteoconductive and osteoinductive non-crystalline materials which tend to form hydroxy carbonate apatite (HCA) when they are in contact with body fluids. The HCA crystalline phase has similar constituents as natural bone, and it is known to interact with and bind to native bone [1]. Although the first discovered BGs consisted of SiO_2_, Na_2_O, CaO, and P_2_O_5_ [2], several types of BGs have been developed using different proportions of silica, phosphorus pentoxide, and boron oxide, which are mainly network formers [3,4]. In general, the amount of silica in silicate glasses ranges from 45–71 wt.%. Other components such as alkali and alkaline earth oxides are used in various proportions as network modifiers [4,5,6,7].

Before the discovery of the sol-gel process, BGs were synthesized by mixing and melting different proportions of the network formers and network modifiers at high temperatures ranging from 1300–1500 °C, followed by quenching in water or on a cold metallic surface [2]. Sol-gel derived BGs can be synthesized using another low-temperature route by colloidal suspension of metal alkoxide precursors in a water/alcohol solution to undergo hydrolysis and polycondensation reactions to form a gel [8,9]. All the constituents of the gel are chemically linked together and uniformly distributed to form a monolithic glass following the drying process. Metal alkoxides, such as tetraethyl orthosilicate (TEOS) or tetramethyl orthosilicate (TMOS), are frequently utilized as SiO_2_ precursors while triethyl phosphate (TEP) is used as a P_2_O_5_ precursor due to their abilities to readily react with water in the hydrolysis stage. Both acid- and base-catalyzed hydrolysis reactions result in the replacement of alkoxy side chains with hydroxyl groups through a nucleophilic attack on the core atom (e.g., Si) by the oxygen atom in the water molecule [8]. The microstructure of the final inorganic glass networks depends on reaction conditions such as pH, acid or base catalysts, the water–reactant ratios, and the precursor molecules [10]. Sol-gel-derived BGs exhibit higher purity, homogeneity, nanoporosity and specific surface area (SSA) compared with melt-derived glasses [11]. Furthermore, compared to melt-derived glasses, sol-gel-derived glasses have more polymerized structures, thus adding to their benefit [12,13].

The increased nanoporosity and surface area of the sol-gel-derived BGs allow for their improved cellular response and bioresorbability [14]. Recently, rod- or wire-shaped biomaterial nanoparticles gained attention due to their larger surface area and ability to reinforce nanocomposites when combined with biopolymers [15,16,17]. Despite their advantages, there are only limited studies that have been conducted to prepare BG nanorods or nanowires [18]. Nanostructure bioceramics can be synthesized by using several techniques such as coprecipitation [19] and sonochemical [20] or hydrothermal processing [18]. Hydrothermal processing has greater advantages than the other methods due to its lower processing temperature and good reproducibility. Hydrothermal processing is a heterogeneous reaction in the presence of aqueous solvents under high pressure and temperature in order to dissolve and re-precipitate the materials at a certain crystallographic axis, which cannot be achieved under ordinary conditions [21]. As a safe and cheap solvent, water serves as the pressure-transmitting medium in the hydrothermal synthesis.

In this study, we first prepared rod-shaped BG nanoparticles using hydrothermal processing at two different temperatures, 130 °C and 170 °C, for various time courses to determine the optimum temperature and reaction time to achieve the best morphology. The synthesized BG nanorods were then blended with different proportions of vinylpyrrolidone (VP) and triethoxyvinylsilane (TEVS), co-polymers with pendant functional groups. In a previous study, we prepared co-polymers with various monomer ratios [22]. Poly(vinyl pyrrolidone) (PVP) is a biocompatible polymer which is approved for use in biomaterials [23,24]. These co-polymers were then mixed with dried BG nanorods in the presence of an ammonium phosphate solution to produce novel bioactive composite biomaterials.

## 2. Materials and Methods

### 2.1. Materials

Tetraethyl orthosilicate (TEOS, 99%), triethyl phosphite (TEP, 98%), calcium nitrate tetrahydrate (CaNO_3_·4H_2_O, 99%), ammonium phosphate dibasic ((NH_4_)_2_HPO_4_, 98%), ammonium dihydrogen phosphate ((NH_4_)H_2_PO_4_, 99%), 1-vinyl-2-pyrrolidone (NVP, 99%), triethoxyvinylsilane (TEVS, 97%), 2,2′-azobis(2-methylpropionitrile) (AIBN, 98%), and phosphate-buffered saline (PBS) were purchased from Sigma-Aldrich (Milwaukee, WI, USA). Anhydrous ethanol was purchased from GreenField Specialty Alcohols Inc. (Chatham, ON, Canada). Petroleum ether was purchased from Caledon Labs (Brampton, ON, Canada).

### 2.2. Sol-Gel Synthesis of Bioactive Glass

Bioactive glass (BG) with a composition of 70 mole% SiO_2_, 26 mole% CaO and 4 mole% P_2_O_5_ was prepared by a sol-gel process in water–ethanol solution. The water to TEOS mole ratio was 4:1. Initially, 1 M HNO_3_ solution was added to the de-ionized (DI) water to reach pH ~3. To prepare 10 g of BG, 1 M HNO_3_ solution was mixed with ethanol (27 mL) at room temperature, followed by adding TEOS (25.6 mL) into the mixture. After 1 h of mixing, 9.85 g Ca(NO_3_)_2_·4H_2_O was added to the solution and mixed until it was dissolved. Then 2.21 mL TEP was added to the mixture and stirred for another 2 h. After that, the sol was transferred to a Teflon mold and covered with tin foil with a few pinholes and kept for 3 days until it gelled at room temperature. The gel was then dried under vacuum at room temperature until transparent glass monoliths were formed. The BG monoliths were then ground into a powder form.

### 2.3. Hydrothermal Processing of Bioactive Glass

**Figure 1:**
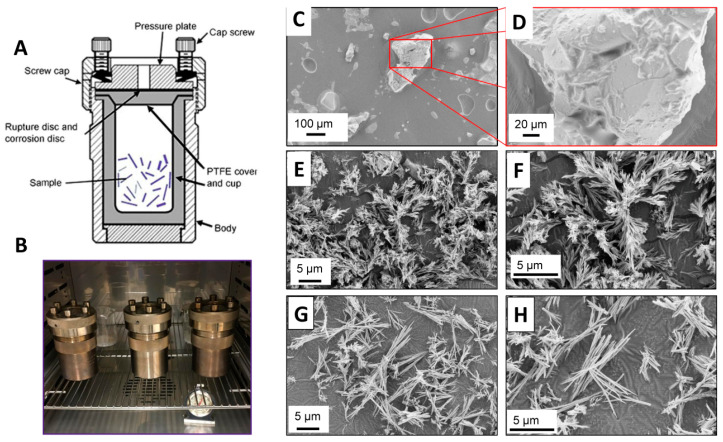
Hydrothermal processing of sol-gel derived tertiary bioactive glass. (**A**) Schematic drawing of a hydrothermal processing unit; (**B**) digital photograph of the hydrothermal units; (**C**,**D**) scanning electron microscopy (SEM) images of as-prepared bioactive glasses by the sol-gel process before hydrothermal processing; (**E**–**H**) representative SEM images of bioactive glasses after 36 h hydrothermal processing at 130 °C (**E**,**F**), and at 170 °C (**G**,**H**).

The hydrothermal processing of BG was conducted at two different temperatures (130 °C and 170 °C) at various times, ranging from 1 h to 36 h, in acid digestion bombs (Figure 1A,B) using aqueous ammonium phosphate solution of pH 7.4. Aqueous solutions used as suspension media were selected to achieve the desired pH. Aqueous solutions included deionized H_2_O, (NH_4_)_2_HPO_4_, and (NH_4_)H_2_PO_4_. One gram of the dried BG powder was suspended in 30 mL solution and vigorously stirred for 30 min. Suspensions were ultrasonically treated for 5 min and vigorously stirred for 30 min. Suspensions were then poured into a 125 mL Teflon-lined acid digestion bomb (Parr Instrumentation Company, Moline, IL, USA), placed into a forced-air oven and heated up to the desired temperature. For each temperature, the duration of hydrothermal treatment was 1, 3, 6, 12, 18, 24 and 36 h. At the end of each time period, the bomb was cooled to 25 °C and the resulting BG nanoparticles were recovered by decanting the aqueous solvent. The particles were collected, washed with deionized H_2_O several times, and centrifuged to produce a pellet. The recovered pellet was then dried under vacuum at room temperature for 24 h.

### 2.4. Synthesis of Co-Polymer

The synthesis of the poly(vinylpyrrolidone-co-vinylsilane) co-polymer hereinafter called poly(VP-co-TEVS) was described in detail in our previous publication [22]. Briefly, stoichiometric amounts of purified NVP and TEVS were mixed in a three-necked flask with a magnetic stirrer bar at 50 °C under N_2_ atmosphere using anhydrous ethanol as a solvent and AIBN as an initiator (0.4 mol % of total NVP and TEVS). The flask was connected to a condenser to reflux the ethanol. Reaction times for co-polymers with varying monomer ratios were optimized based on maintaining a consistent range of weight-average molecular weights (M_w_) (35–45 kDa). Purification of the co-polymers was carried out by repeated precipitation in excess (volume ≥10 times than the co-polymer solution) cold petroleum ether. The precipitated co-polymer was dissolved in ethanol and re-precipitated again in petroleum ether three times. Finally, the polymer was dissolved in ethanol, followed by transferring it into a polytetrafluoroethylene (PTFE) beaker and placed into a vacuum dryer at room temperature for 24 h.

### 2.5. Synthesis of Bioactive Composite Biomaterials

The liquid component was prepared as follows: 60.1 g reagent-grade (NH_4_)_2_HPO_4_ and 5.0 g NH_4_H_2_PO_4_ were dissolved into 100 mL of DI water to form an ammonium phosphate solution of pH 7.4. The composite materials were prepared as follows: different proportions of BG nanorods and cryo-milled poly(VP-co-TEVS) and the ammonium phosphate liquid were mixed homogeneously on a glass plate with a powder to liquid ratio of 0.8 until consistency was achieved. The paste was then loaded into a 1 mL syringe and left to harden for 24 h.

### 2.6. Scanning Electron Microscopy (SEM), X-ray Diffraction (XRD), and Fourier Transform Infrared Spectroscopy (FTIR)

The morphology of BG nanorods was visualized using a Zeiss Leo 1540XB SEM (Carl Zeiss, Oberkochen, Germany) at 5 mm working distance and 3 kV of electron beam voltage. The specimens were coated with Osmium in an Osmium Plasma Coater (OPC80T, Filgen Inc., Nagoya, Japan). Energy Dispersive X-ray (EDX) instrument attached to the SEM was used for elemental analysis. XRD was performed using an X-ray diffractometer AXS D2 PHASER (Bruker Corporation, Billerica, MA, USA) operating on CuKα radiation with λ = 1.5418Å. The measurements were conducted in 30 kV and 10 mA in 2θ range 10–60° with steps of 0.049°. For FTIR analysis, BG nanorods were ground in a planetary ball mill to get a fine powder. FTIR spectra of this powder were obtained using a Nicolet 6700 FT-IR Spectrometer (Thermo Scientific, Waltham, MA, USA) at a resolution of 4 cm^−1^ with a sample scan of 32 to identify specific functional groups. All spectra were analyzed using OMNIC series software.

### 2.7. Solid-State Cross-Polarization Magic-Angle Spinning (CPMAS) ^29^Si NMR

^29^Si NMR spectra of hydrothermally treated BG and the composite were acquired using a Varian Infinity Plus 400 NMR spectrometer (ν(^1^H) = 399.5 MHz, ν(^29^Si) = 79.4 MHz) equipped with a Varian triple-resonance (H-X-Y) 7.5 mm magic-angle spinning NMR probe. The samples were packed tightly into 7.5 mm outer diameter ZrO_2_ rotors and rotated at 5.5 kHz. A total of 4000 scans were summed using a 6.75 μs ^1^H 90-degree pulse, 2 ms contact time, 10.24 ms acquisition time, 7 s recycle delay, 50 kHz spectral width and continuous-wave ^1^H decoupling during acquisition. For processing, two zero-fills and 30 Hz line broadening were applied to the free induction decay before Fourier transformation. The NMR spectra were referenced with respect to tetramethylsilane (δ(^29^Si) = 0.0 ppm) by setting the high-frequency peak of tetrakis(trimethylsilyl)silane to −9.8 ppm.

### 2.8. Mechanical and Degradation Testing of Poly(VP-co-TEVS)/BG Composites

The hardened composite loaded in a 1 ml syringe was cut using a diamond blade into 10 cylindrical specimens of 3 mm in diameter and 5 mm in height. Compression testing of these specimens was conducted using an Instron universal machine, model number 3345 (Norwood, MA, USA), at a crosshead speed of 0.1 mm/min with 5 kN (10% and 30% samples) and 500 N (0% samples) load cells. The compressive elastic modulus and fracture strength of each specimen was recorded. Degradation behavior was characterized by studying the weight loss and change in morphology of the poly(VP-co-TEVS)/BG composite following incubation in PBS for various times. Disk specimens (6 mm in diameter and 2 mm in thickness) of composites were weighed (initial weight) and then incubated in 30 mL PBS solution in polypropylene bottles covered with a tight lid. The bottles were placed in an orbital shaker at 120 rpm and 37 °C and incubated for 1, 3, 6, 9, 12 or 15 days. After each time point, specimens were removed and rinsed with deionized water, dried under vacuum at room temperature for 24 h, and weighed (final weight). The percentage weight loss for each specimen was calculated from its initial and final weight.

### 2.9. MC3T3-E1 Preosteoblast Culture

Cell culture was conducted by seeding MC3T3-E1 pre-osteoblastic cells (Subclone 4, American Type Culture Collection, Manassas, VA, USA) onto control glass slides, polymethylmethacrylate (PMMA) controls and composite disks. MC3T3-E1 cells were cultured in standard tissue culture plates using α-MEM supplemented with 10% fetal bovine serum (FBS) and 1% antibiotic–antimycotic solution. The medium was changed every 2 days and cells were removed from the plates using trypsin–ethylenediaminetetraacetic acid solution. The hardened composite loaded into a 3 mL syringe was cut using a diamond blade into 9 disk specimens of 6 mm in diameter and 1.5 mm in height. Disks were incubated in media solution for 1 week to make sure that the pH was 7.4 prior to cell seeding. All disks were disinfected by submerging them into 70 vol % ethanol for 1 h and dried. Prior to seeding the cells, each disk was incubated in 1 mL serum-free culture medium. The disks were transferred to 48 well plates and MC3T3-E1 pre-osteoblastic cells were seeded at a density of 10,000 cells/well and incubated for 6 h, 7 days and 14 days. The culture medium was changed every 2 days. Specimens were fixed at 6, 24 and 48 h from seeding and subsequently blocked and stained with phalloidin for F-actin and 4′,6-diamidino-2-phenylindole (DAPI) for nuclei.

## 3. Results and Discussion

### 3.1. Hydrothermal Processing of Bioactive Glass

Hydrothermal processing refers to a series of heterogeneous reactions in the presence of aqueous solvents under high pressure and temperature in order to dissolve and recrystallize materials which are fairly insoluble under normal conditions [21]. Water acts as the solvent and the pressure-conveying medium in hydrothermal processing. Under hydrothermal conditions, the ionic product of water increases with higher pressure and temperature, and the viscosity decreases. The low viscosity and high reactivity allow it to be excellent reaction media for the synthesis of metastable phases and the growth of microcrystallites with well-defined size and morphology for specific applications. In the current study, the rod-shaped BG nanoparticles were successfully synthesized using hydrothermal processing at 130 °C and 170 °C for various times. Figure 1C–H shows representative SEM images of as-prepared BG particles with hydrothermally-treated BG at two different temperatures. The sol-gel derived and ground BG particles were large and exhibited irregular morphology. After hydrothermal processing at 130 °C for 36 h, these large, micron-sized, irregularly shaped particles turned into dandelion or broom-like nanoparticles (Figure 1F). These particles may have been formed by a mechanism of crystal splitting [25], and it has been suggested that rapid crystal growth in oversaturated solution may initiate this phenomenon. During hydrothermal processing, rapid dissolution and precipitation occur under elevated temperature and pressure, which induce the nucleation and growth of single crystals. Hence, after hydrothermal processing of the BG particles at 170 °C for 36 h, a dramatic increment in the nanoparticle length occurred (Figure 1H). It is clearly visible that increasing the temperature from 130 °C to 170 °C enhanced the growth of these nanoparticles in a single preferred direction.

### 3.2. The Effect of Reaction Time on Morphology and Crystallinity

**Figure 2:**
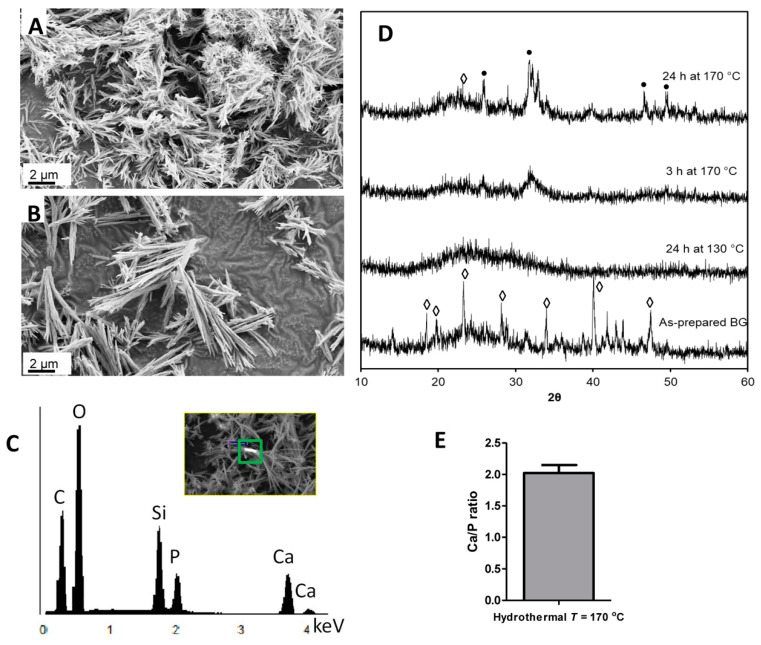
The effect of hydrothermal processing time on the morphological evolution of bioactive glass (BG). (**A**) 170 °C for 3 h and (**B**) 170 °C for 24 h. (**C**) EDX spectra of nanorods prepared at 170 °C for 24 h. The region shown by the green square in the inset in Figure C was used to generate the EDX spectra. (**D**) XRD patterns of as-prepared and hydrothermally-processed BG; (•) indicates hydroxyapatite and (◊) SiO_2_. (**E**) Ca/P ratio of nanorods prepared at 170 °C for 24 h.

The SEM images of BG nanoparticles subjected to hydrothermal processing for 3 and 24 h at 170 °C are shown in Figure 2A,B. The rod-shaped morphology evolved from nucleation to splitting and, finally, growth. At 3 h, the nanoparticles started to split and grow in several directions (Figure 2A). These short and spindle-shaped nanoparticles were aggregated and grown into rod-shaped particles at longer reaction times (Figure 2B). The EDX data (Figure 2C) showed that the tertiary BG contained the expected elements of calcium, silicon, and phosphorus. The oxygen is from CaO and P_2_O_5_; however, the carbon is from the SEM stub and not from the BG since the as-prepared material has been fully hydrolyzed and carbon entrapment within the synthesized material was not expected. The entrapment of carbon will cause the as-prepared material to turn into a greyish or black color, which was not observed. XRD profiles of the nanoparticles clearly depicted the crystal formation under hydrothermal conditions (Figure 2D). The as-prepared sol-gel-derived BG particles consisted of crystalline phases of SiO_2_. However, 24 h of reaction time under hydrothermal conditions at 130 °C converted the sol-gel-derived BG entirely into amorphous BG glass. Interestingly, as the nucleation and splitting were taking place at 170 °C for 3 h under hydrothermal conditions, the crystalline hydroxyapatite phase started to form. After 24 h, more matured crystalline phases of hydroxyapatite were induced. Thus, the transition from amorphous BG glass to glass–ceramic was influenced by the longer reaction time of the hydrothermal processing.

**Figure 3:**
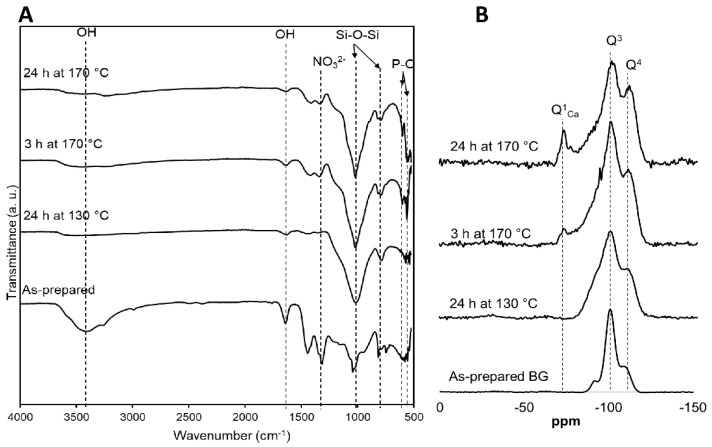
(**A**) FTIR spectra and (**B**) solid-state Si-NMR of sol-gel-derived and hydrothermal processing of BG glasses at 130 °C and 170 °C for 3 hrs and 24 h. Q represents the structure of bridging networks of BG at post-processing. Q^4^ indicates the Si is bonded with 4 -O-Si-, whereas Q^1^ indicates the Si is bonded with one -O-Si and three -OH. Q^1^_Ca_ is associated with the peak for Q^1^ structure, where at least one -OH is replaced by calcium.

We have also evaluated the atomic Ca/P ratio of the hydrothermal treated BGs and it was 2.02 ± 0.13 (Figure 2E). Since the stoichiometric atomic Ca/P ratio in hydroxyapatite is 1.67, it suggests that we had more calcium and that the synthesized tertiary glass–ceramic following hydrothermal processing at 170 °C for 24 h consists of mixed phases including CaO, SiO_2_ and HA. Sol-gel-derived, and subsequently, calcined tertiary glasses had shown an atomic Ca/P ratio between 1.67 and 1.77 when the calcination temperature was at or below 600 °C [26,27]. Increasing the calcination temperature above 600 °C resulted in an increased Ca/P ratio due to the formation of CaO along with hydroxyapatite [26]. The bioactive glasses in the current study were not calcined; instead, hydrothermal processing was used at a temperature of 130–170 °C. As shown in the FTIR spectra (Figure 3), the nitrate groups progressively vanished as the hydrothermal temperature was increased to 170 °C.

### 3.3. Characteristics of Chemical Network Formation as a Result of Hydrothermal Processing

Figure 3A displays the FTIR transmittance curves of the BG nanoparticles prepared at different temperatures and time courses. For both the sol-gel-derived and hydrothermally-processed BGs, the bands for Si-O-Si symmetric stretching and Si-O-Si asymmetric stretching were visible at 787 cm^−1^ and at 1020 cm^−1^, respectively [28]. Broader bands were observed at 3423 and 1614 cm^−1^ due to the stretching vibration of the -OH from the non-bridging oxygens of the BG network. FTIR spectra of BG treated at 170 °C showed bands at 1350 cm^−1^ related to nitrate ions, which were usually present in sol-gel-derived BG glasses as a result of using HNO_3_ as a catalyst in the synthesis procedure [29]. The elimination of bands at 966 cm^−1^ in the FTIR spectra of hydrothermally-processed BG associated with non-bridging oxygens indicated that the crystalline phases of BG appeared due to the hydrothermal processing and is consistent with the results from XRD analysis. Bands at 603 and 557 cm^−1^ are associated with -P-O, and they appeared after treatment at 170 °C for 24 h. This result indicates the incorporation of phosphate into the glass network.

Figure 3B displays the ^29^Si-NMR spectra of the sol-gel-derived and hydrothermally-processed BG particles. All the specimens, before and after hydrothermal processing, exhibited dominant Q^3^ and Q^4^ species. Q*^n^* corresponds to the structures of Si-O-Si(OSi-)*_n_*(OH)*_4-n_*, where OH is referred to as non-bridging oxygens. Chemical shifts at approximately −100 and −110 ppm are associated with Q^3^ and Q^4^ species, respectively. In addition to Q^3^ and Q^4^ structures, there were peaks observed at −73 ppm for BGs processed at 170 °C, which are associated with Q^1^_Ca_ structures (Si-O-Si networks containing three bridging oxygens in which one of the H was replaced by calcium (-Si-O-Ca)) [30,31,32]. It has been previously reported that BGs are required to be heated to a temperature greater than 400 °C to incorporate Ca into the glass networks [1]. In this study, we found that a weak peak was visible for Q^1^_Ca_ following 3 h of hydrothermal processing at 170 °C. Furthermore, a strong peak for Q^1^_Ca_ was visible after heat treatment for 24 h. This finding suggests that the hydrothermal conditions enhanced the formation of calcium incorporated into the glass network.

### 3.4. Properties of Composites Prepared with BG Nanorods and Synthesized Co-Polymers

**Figure 4:**
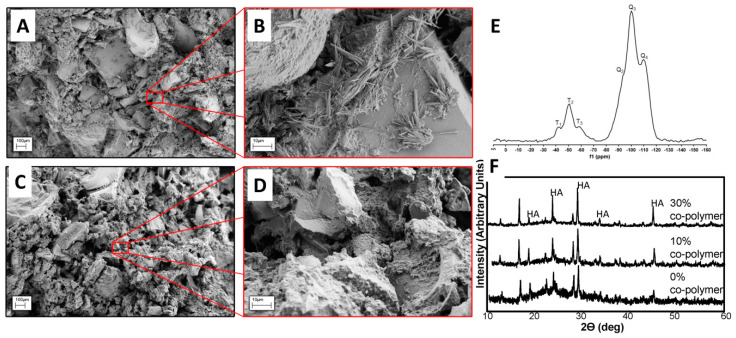
SEM images of composites consisting of hydrothermally-treated (at 170 °C for 24 h) BG nanorods without a co-polymer (**A**,**B**) and composites made of hydrothermally-treated (at 170 °C for 24 h) BG nanorods with a co-polymer (**C**,**D**). Solid-state Si-NMR of hydrothermally treated bioactive glass composites (**E**) and XRD profiles of the bioactive composites showed HA formation (**F**).

Aqueous solutions of poly(VP-co-TEVS) and ammonium phosphate were mixed with BG nanorods to prepare the bioactive composites. Composites made without co-polymer were also prepared as controls. Representative SEM images of the surface of the as-prepared composites without co-polymer are shown in Figure 4A,D and they exhibited large micron-sized hydroxyapatite (HA) particles and irregular morphologies (Figure 4A,B). BG nanorods were seen loosely dispersed on the surfaces of HA particles. The hardening process of BG nanorods with ammonium phosphate (the liquid component) involves dissolution and reprecipitation. During dissolution, the nanorod glass–ceramics release calcium and phosphate ions, generating supersaturation in the solution [33]. Following this, the nucleation of the new phase starts and continues growing, generally surrounding the nanorod particles (Figure 4A,B) through the well-known acid–base chemistry of the calcium phosphate hardening reaction [33,34]. Thus, even without adding the poly(VP-co-TEVS), it is already a composite of BG ceramic/calcium phosphate precipitate matrix. The addition of co-polymer into the composites enhanced the dispersion of the nanorods in a controlled manner with more regular morphologies (Figure 4C,D). Moreover, the BG nanorods were embedded inside the composites, which may provide superior mechanical strength and controlled degradation behavior. Solid-state Si-NMR has shown the formation of T^n^ structures which indicates covalent crosslinking between the co-polymers and BG nanorods [22,35]. XRD profiles have shown HA formation during the setting of the composites (Figure 4E).

**Figure 5:**
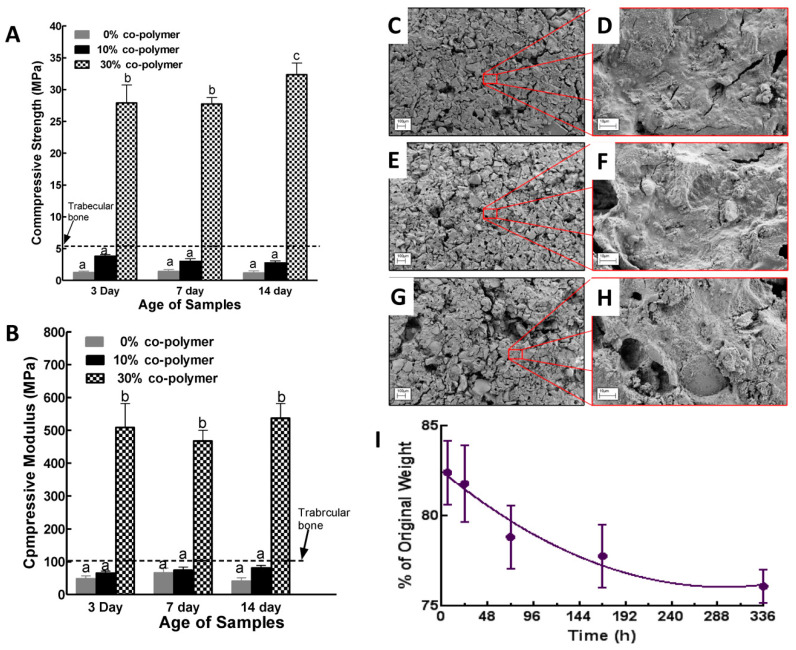
Compressive mechanical behavior and degradation profiles of bioactive composite materials. (**A**) Cylindrical bioactive composite ultimate compressive strength, (**B**) and compressive moduli. Data are means ± standard deviation (SD) (n = 8). Two-way ANOVA and Tukey’s multiple comparison tests were used for statistical analysis. Different lower-case letters indicate significance at *p* < 0.05. (**C**–**H**) SEM images of bioactive composites degraded in phosphate-buffered saline (PBS) at different times. Composite made of hydrothermally-processed (at 170 °C for 24 h) bioactive glass nanorods and 30 wt % co-polymer. Surfaces of composite disks following PBS soaking for 3 days (**C**,**D**), 7 days (**E**,**F**), and 14 days (**G**,**H**). (**I**) Weight loss over the time courses. Data are means ± SD (n = 5). Two-way ANOVA and Tukey’s multiple comparison tests were used for statistical analysis.

Composites with covalent crosslinking between polymers and BGs are known to provide better mechanical properties and more controlled degradation behavior when compared to conventional composites without strong interaction within the different phases [36]. In this study, the addition of 10% co-polymer did not affect the compressive strength and modulus significantly compared to the composite made without co-polymer. However, the addition of 30% co-polymer resulted in an increase in the strength and modulus values significantly (*p* < 0.01; Figure 5A,B). These co-polymers used in the present study had pendant trialkoxysilane functional groups as side chains, which provided a much higher number of reactive sites to BG nanorods during composite formation. Incorporation of a higher amount of co-polymer increased the degree of crosslinking, which enhanced the mechanical properties of the composites. The compressive mechanical properties data were consistent with the solid-state Si-NMR results.

**Figure 6:**
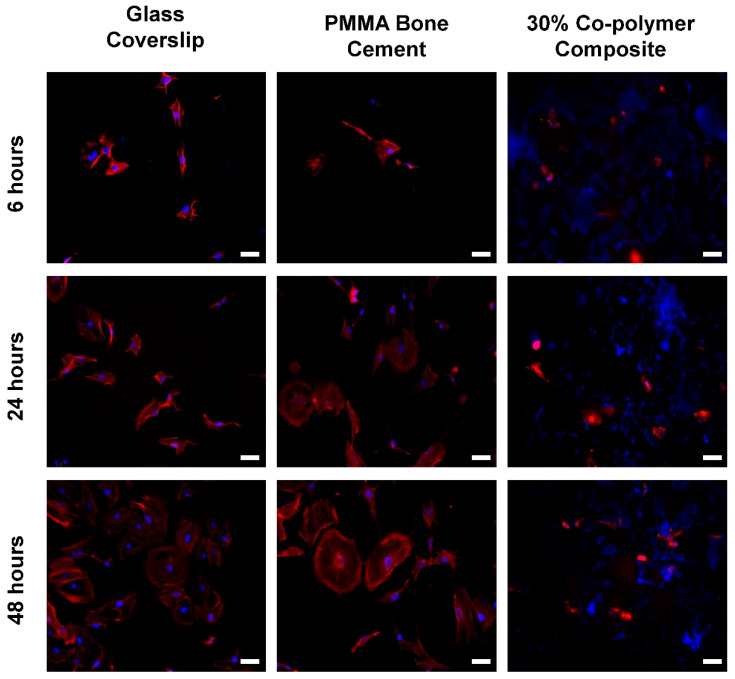
MC3T3-E1 cell adhesion on glass coverslips, PMMA disks and poly(VP-co-TEVS)-BG ceramic composites. Scale bar = 40 μm. Blue is nuclei, and red is F-actin.

The degradation of composites made with BG nanorods was studied by evaluating weight loss and examining the surface morphologies after incubating in PBS over a period of 1–15 days (Figure 5C–I). The specimens lost around 17% of their initial weight after just a few hours of incubation. This was due to the fact that some unreacted constituents such as BG nanorods, ammonium phosphate and co-polymers which were entrapped in the composite materials were leaching out. Degradation of the composite was more controlled from one to seven days. Weight loss between 7 to 14 days was not significantly different, which suggests the stability of the composite formulation beyond 25% mass loss. The cytocompatibility of the synthesized poly(VP-co-TEVS)-BG composites was investigated by assessing the morphology and spreading of preosteoblastic MC3T3-E1 cells. Cells were examined 6, 24 and 48 h following seeding on a glass coverslip, PMMA disks and composite surfaces. Cells were fixed and labeled for F-actin and DNA (red and blue respectively in Figure 6). At 6 h, cells started to adhere to composite surfaces. However, after 24 h and 48 h, cells were not readily spreading on composite surfaces compared to PMMA and glass coverslips. This behavior could be due to the release of unleached materials even after preconditioning the composites for seven days to stabilize the pH. Another possibility is the lack of surface modification by adsorbed cell adhesive proteins (e.g., fibronectin) to promote cell attachment and spreading. Yet another possible reason could be the surface morphology of the composite possibly having an effect. These potential reasons will be investigated in future studies.

In this study, we reported a composite biomaterial prepared from hydrothermally-treated bioactive glass–ceramic and poly(VP-co-TEVS) which was further modified with ammonium phosphate. Hydrothermal processing at a relatively low temperature (150 °C) is a versatile process to create bioactive glass–ceramics with interesting morphologies. It is reported in the literature that bioactive glasses of compositions 58SiO_2_-33CaO-9P_2_O_5_ and 60SiO_2_-30CaO-10P_2_O_5_ were synthesized by the sol-gel process, whereby sols were hydrothermally processed at temperatures ranging from 150 to 220 °C to obtain varieties of nanoparticles and nanorods [18,37]. However, our approach is different from these cited studies. In the present study, a bioactive glass whose composition is 70 mole % SiO_2_, 26 mole% CaO and 4 mole% P_2_O_5_ was first synthesized until the sol has completely gelled. The gels were then dried and hydrothermally processed at temperatures ranging from 130 to 170 °C in the presence of ammonium hydroxide. This base-induced dissolution and reprecipitation resulted in a nanorod morphology while, at the same time, generating additional OH groups on these nanorod bioactive glass–ceramic surfaces. Finally, in the presence of ammonium phosphate and poly(VP-co-TEVS), the nanorod bioactive glass–ceramic underwent a combination of an acid–base reaction to form CaNH_4_PO_4_.H_2_O and further crosslinking with the co-polymer functional groups to produce bioactive glass–ceramic co-polymer composites. We showed that the compressive mechanical properties were significantly improved by the presence of poly(VP-co-TEVS) and depended on the amount added. Crosslinking the nanorod bioactive glass–ceramics with ammonium phosphate alone resulted in a weak compressive strength and modulus. However, the enhanced compressive mechanical properties with 30% co-polymer incorporation are comparable to reported bone cement formulation from melt-derived bioactive glasses [38,39,40]. Interestingly, the compressive mechanical properties of the composites made from hydrothermally-processed bioactive glass–ceramics without co-polymer (i.e., 0% co-polymer in Figure 5A,B) were lower than the reported values from melt-derived bioactive glasses. This difference is likely due to the quenching process in melt-derived glasses that will make it amorphous and hence a stronger acid–base reaction will lead to higher mechanical properties.

## 4. Conclusions

Rod-shaped ternary bioactive glass nanoparticles were prepared successfully by using hydrothermal processing at 170 °C. The morphology, shape and chemical properties of these nanorods were dependent on processing temperature and duration. Hydrothermal processing of sol-gel derived BG at 170 °C for 24 h produced uniformly distributed nanorods with incorporated calcium into the glass matrices. This method has shown to be a facile way to incorporate calcium into the glass network compared to the conventional technique which required the calcination of glass powders above 600 °C. The nanorods were successfully combined with poly(VP-co-TEVS) and ammonium phosphate to produce bioactive composites. These newly-developed bioactive materials have shown improved mechanical properties and controlled degradation behavior. Fibroblast cell attachment and spreading on the composite were not as good as the positive control surfaces, which suggested they may require protein coating in order to promote favorable cell interactions. The biomaterials have the potential to be used as grafts for bone regeneration.

## References

[1] Aslankoohi N., Mondal D., Rizkalla A.S., Mequanint K. (2019). Bone repair and regenerative biomaterials: Towards recapitulating the microenvironment. Polymers.

[2] Hench L.L., Splinter R.J., Allen W.C., Greenlee T.K. (1971). Bonding mechanisms at the interface of ceramic prosthetic materials. J. Biomed. Mater. Res..

[3] Kaur G., Pandey O.P., Singh K., Homa D., Scott B., Pickrell G. (2013). A review of bioactive glasses: Their structure, properties, fabrication and apatite formation. J. Biomed. Mater. Res. Part A.

[4] Rahaman M.N., Day D.E., Sonny Bal B., Fu Q., Jung S.B., Bonewald L.F., Tomsia A.P. (2011). Bioactive glass in tissue engineering. Acta Biomater..

[5] Brink M. (1998). The influence of alkali and alkaline earths on the working range for bioactive glasses. J. Biomed. Mater. Res..

[6] Brovarone C.V., Verné E., Appendino P. (2006). Macroporous bioactive glass-ceramic scaffolds for tissue engineering. J. Mater. Sci. Mater. Med..

[7] Fu Q., Rahaman M.N., Sonny Bal B., Brown R.F., Day D.E. (2008). Mechanical and in vitro performance of 13–93 bioactive glass scaffolds prepared by a polymer foam replication technique. Acta Biomater..

[8] Hench L.L., West J.K. (1990). The sol-gel process. Chem. Rev..

[9] Allo B.A., Rizkalla A.S., Mequanint K. (2010). Synthesis and electrospinning of ε-polycaprolactone-bioactive glass hybrid biomaterials via a sol−gel process. Langmuir.

[10] Brinker C.J. (1988). Hydrolysis and condensation of silicates: Effects on structure. J. Non-Cryst. Solids.

[11] Sepulveda P., Jones J.R., Hench L.L. (2002). Characterization of melt-derived 45S5 and sol-gel–derived 58S bioactive glasses. J. Biomed. Mater. Res..

[12] Dziadek M., Zagrajczuk B., Jelen P., Olejniczak Z., Cholewa-Kowalska K. (2016). Structural variations of bioactive glasses obtained by different synthesis routes. Ceram. Int..

[13] Wajda A., Sitarz M. (2018). Structural and microstructural comparison of bioactive melt-derived and gel-derived glasses from CaO-SiO_2_ binary system. Ceram. Int..

[14] Lei B., Chen X., Wang Y., Zhao N., Du C., Fang L. (2010). Surface nanoscale patterning of bioactive glass to support cellular growth and differentiation. J. Biomed. Mater. Res. Part A.

[15] Azizian M., Rabiee S.M. (2016). A Freestanding Sol-Gel Technique for Growth of Nanowire Arrays in SiO_2_-CaO-P_2_O_5_-ZrO_2_ System. Silicon.

[16] Rabiee S.M., Azizian M. (2013). Effect of Zirconia Concentration on the Growth of Nanowires in Bioactive Glass–Ceramic Coatings. Int. J. Appl. Ceram. Technol..

[17] Rahong S., Yasui T., Kaji N., Baba Y. (2016). Recent developments in nanowires for bio-applications from molecular to cellular levels. Lab. Chip.

[18] Zeimaran E., Pourshahrestani S., Shirazi S.F.S., Pingguan-Murphy B., Kadri N.A., Towler M.R. (2016). Hydrothermal synthesis and characterisation of bioactive glass-ceramic nanorods. J. Non-Cryst. Solids.

[19] Hong Z., Merino E.G., Reis R.L., Mano J.F. (2009). Novel Rice-shaped Bioactive Ceramic Nanoparticles. Adv. Eng. Mater..

[20] Qi C., Zhu Y.-J., Zhao X.-Y., Zhao J., Chen F., Cheng G.-F., Ruan Y.J. (2013). High surface area carbonate apatite nanorod bundles: Surfactant-free sonochemical synthesis and drug loading and release properties. Mater. Res. Bull..

[21] Byrappa K., Yoshimura M., Byrappa K., Yoshimura M. (2001). 1—Hydrothermal Technology—Principles and Applications. Handbook of Hydrothermal Technology.

[22] Mondal D., Dixon S.J., Mequanint K., Rizkalla A.S. (2018). Bioactivity, Degradation, and Mechanical Properties of Poly(vinylpyrrolidone-co-triethoxyvinylsilane)/Tertiary Bioactive Glass Hybrids. ACS Appl. Bio Mater..

[23] Karageorgiou V., Kaplan D. (2005). Porosity of 3D biomaterial scaffolds and osteogenesis. Biomaterials.

[24] Teodorescu M., Bercea M. (2015). Poly(vinylpyrrolidone)—A Versatile Polymer for Biomedical and Beyond Medical Applications. Polym.-Plast. Technol. Eng..

[25] Tang J., Alivisatos A.P. (2006). Crystal Splitting in the Growth of Bi_2_S_3_. Nano Lett..

[26] Sanosh K.P., Chu M.C., Balakrishnan A., Kim T.N., Cho S.J. (2009). Preparation and characterization of nano-hydroxyapatite powder using sol-gel technique. B Mater. Sci..

[27] Gentile P., Wilcock C.J., Miller C.A., Moorehead R., Hatton P.V. (2015). Process Optimisation to Control the Physico-Chemical Characteristics of Biomimetic Nanoscale Hydroxyapatites Prepared Using Wet Chemical Precipitation. Materials.

[28] Ma J., Chen C.Z., Wang D.G., Meng X.G., Shi J.Z. (2010). Influence of the sintering temperature on the structural feature and bioactivity of sol–gel derived SiO_2_–CaO–P_2_O_5_ bioglass. Ceram. Int..

[29] Catauro M., Bollino F., Renella R.A., Papale F. (2015). Sol–gel synthesis of SiO2–CaO–P2O5 glasses: Influence of the heat treatment on their bioactivity and biocompatibility. Ceram. Int..

[30] Gunawidjaja P.N., Mathew R., Lo A.Y.H., Izquierdo-Barba I., García A., Arcos D., Vallet-Regí M., Edén M. (2012). Local structures of mesoporous bioactive glasses and their surface alterations in vitro: Inferences from solid-state nuclear magnetic resonance. Philos. Trans. A Math. Phys. Eng. Sci..

[31] Martin R.A., Twyman H.L., Rees G.J., Smith J.M., Barney E.R., Smith M.E., Hanna J.V., Newport R.J. (2012). A structural investigation of the alkali metal site distribution within bioactive glass using neutron diffraction and multinuclear solid state NMR. Phys. Chem. Chem. Phys..

[32] Pedone A., Charpentier T., Malavasi G., Menziani M.C. (2010). New Insights into the Atomic Structure of 45S5 Bioglass by Means of Solid-State NMR Spectroscopy and Accurate First-Principles Simulations. Chem. Mater..

[33] Zhang J., Liu W., Schnitzler V., Tancret F., Bouler J.M. (2014). Calcium phosphate cements for bone substitution: Chemistry, handling and mechanical properties. Acta Biomater..

[34] Fernandez E., Gil F.J., Ginebra M.P., Driessens F.C., Planell J.A., Best S.M. (1999). Calcium phosphate bone cements for clinical applications. Part II: Precipitate formation during setting reactions. J. Mater. Sci. Mater. Med..

[35] Mondal D., Rizkalla A.S., Mequanint K. (2016). Bioactive borophosphosilicate-polycaprolactone hybrid biomaterials via a non--aqueous sol gel process. RSC Adv..

[36] Mondal D., Dixon S.J., Mequanint K., Rizkalla A.S. (2017). Mechanically-competent and cytocompatible polycaprolactone-borophosphosilicate hybrid biomaterials. J. Mech. Behav. Biomed. Mater..

[37] Bui V.X., Vo M.Q., Nguyen T.A., Bui H.T. (2019). Investigation of bioactive glass-cermaic 60SiO_2_-30CaO-10P_2_O_5_ prepared by hydrothermal method. Adv. Mater. Sci. Eng..

[38] Taguchi Y., Yamamuro T., Nakamura T., Nishimura N., Kokubo T., Takahata E., Yoshihara S. (1990). A bioactive glass powder-ammonium hydrogen phosphate composite for repairing bone defects. J. Appl. Biomater..

[39] Nishimura N., Yamamuro T., Taguchi Y., Ikenaga M., Nakamura T., Kokubo T., Yoshihara S. (1991). A new bioactive bone cement: Its histological and mechanical characterization. J. Appl. Biomater..

[40] Kokubo T., Yoshihara S., Nishimura N., Yamamuro T., Nakamura T. (1991). Bioactive Bone Cement Based on Ca-SiO_2_-P_2_O_5_ Glass. J. Am. Ceram. Soc..

